# Phytochemicals and PI3K Inhibitors in Cancer—An Insight

**DOI:** 10.3389/fphar.2017.00916

**Published:** 2017-12-14

**Authors:** Vasanti Suvarna, Manikanta Murahari, Tabassum Khan, Pramila Chaubey, Preeti Sangave

**Affiliations:** ^1^Department of Pharmaceutical Chemistry and Quality Assurance, SVKM's Dr. Bhanuben Nanavati College of Pharmacy, Mumbai, India; ^2^Department of Pharmaceutical Chemistry, Faculty of Pharmacy, M.S Ramaiah University of Applied Sciences, Bangalore, India; ^3^Department of Pharmaceutics, SVKM's Dr. Bhanuben Nanavati College of Pharmacy, Mumbai, India; ^4^Department of Pharmaceutical Sciences, School of Pharmacy and Technology Management, SVKM's NMIMS, Mumbai, India

**Keywords:** cancer, clinical trials, phytochemicals, Mitogen-activated protein kinases (MAPK), Mammalian TORC pathway, phosphatidylinositol-4, 5-bisphosphate (PI3K)

## Abstract

In today's world of modern medicine and novel therapies, cancer still remains to be one of the prime contributor to the death of people worldwide. The modern therapies improve condition of cancer patients and are effective in early stages of cancer but the advanced metastasized stage of cancer remains untreatable. Also most of the cancer therapies are expensive and are associated with adverse side effects. Thus, considering the current status of cancer treatment there is scope to search for efficient therapies which are cost-effective and are associated with lesser and milder side effects. Phytochemicals have been utilized for many decades to prevent and cure various ailments and current evidences indicate use of phytochemicals as an effective treatment for cancer. Hyperactivation of phosphoinositide 3-kinase (PI3K) signaling cascades is a common phenomenon in most types of cancers. Thus, natural substances targeting PI3K pathway can be of great therapeutic potential in the treatment of cancer patients. This chapter summarizes the updated research on plant-derived substances targeting PI3K pathway and the current status of their preclinical studies and clinical trials.

## Introduction

Cancer is the primary reason of mortality in developing countries and secondary cause of mortality in third world countries (Meybodi et al., 2017). According to most recent statistics of cancer prevalence and death worldwide provided by the International Agency for Research on Cancer 2012, there are about 14 million new annual incidences of cancer. This number is expected to upsurge to 22 million per year within successive two decades. Developing countries have 60% of the world's total cases of cancer and 70% of world's deaths due to cancer attributing to lack of accessibility to treatment and failure of early detection. This is increasing the economic and social burden due to cancer incidence in such countries. Thus, effective treatment and prevention of cancer is important as cancer continues to be a major health problem worldwide (Jiang and Liu, 2008).

**Figure 1:**
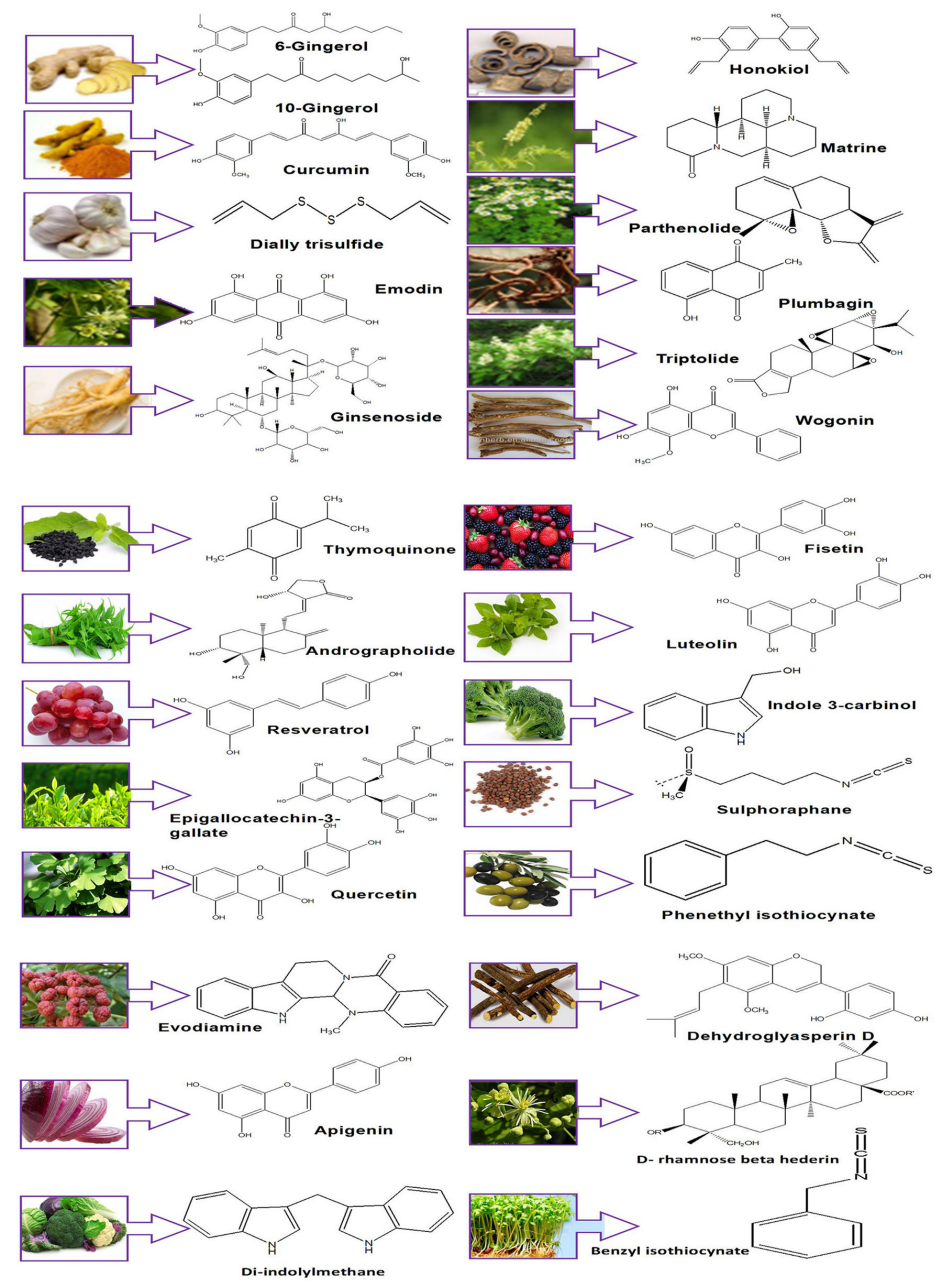
Phytochemicals and their sources.

For the past 30 years, natural and synthetic compounds have been the backbone for cancer chemotherapy (Mann, 2002). However, there are some shortcomings related to synthetic drugs such as associated toxic side effects which limit their use and also the stringent regulatory process which they need to pass before they come in the market. In contrast, natural compounds from dietary sources do not have such toxic side effects and thus they are more appealing than the synthetic compounds. Phytochemicals are the secondary plant metabolites such as triterpenoids, flavonoids, catechols, sulphated carbohydrates, tannins. The literature reveals several natural compounds present in plant extracts, herbs, vegetables, and fruits exhibit anticancer activity against numerous cancers. Also various phytoconstituents have proven to be associated with protective effect in cancer treatment (Michaud et al., 2000). Thus, phytochemicals attract more scientific research due to their wide acceptance and cost-effectiveness. From the 1940s to the early 2010s, almost 70% of the anticancer drugs are non-synthetic and 50% of them being phytochemicals such as vinca alkaloids, taxanes, camptothecins which possess cytotoxic activity contributing to effective cancer treatment (Jiang and Liu, 2008). Figure 1 and Table 1 represents examples of various phytochemicals and their sources proven to have anticancer activity. These compounds have also shown anti-angiogenic effects. Further herbal extracts are investigated in order to identify molecules targeting different pathways of angiogenesis (Kadioglu et al., 2013).

**Table 1:** Chemical and therapeutic profile of phytochemicals.

**Name of compounds**	**Chemical classification**	**Possible mechanism of action**	**Therapeutic uses**
Gingerols	Predominated phenols of ginger oil	Inhibition of PI3K/Akt pathway	Anticancer, antineuroinflammatory, antioxidant, anti-inflammatory, anti-cancer
Curcumin	Flavonoid	Inhibition of PI3K/Akt/mTOR pathway	Cardiovascular diseases, arthritis and inflammation, breast, gastric, colon, prostate cancers, melanoma, lymphoma, and leukemia
Diallyl Trisulfide	Organosulfur compound	Inhibition of PI3K/Akt pathway	Diabetes, cardiovascular disease, stimulate immune system, induce apoptosis in colon, gastric, prostate, and breast cancers
Emodin	Anthraquinone derivative	Inhibition of PI3K/Akt pathway	Several human cancers, neuroprotective, anti-viral
Ginsenoside RG3	Steroid glycosides and triterpene saponins	Inhibition of PI3K/Akt pathway	Numerous types of cancers- ovarian, lung, melanoma
D-Rhamnose β-hederin	Triterpenoid saponin	Inhibition of PI3K/Akt pathway	Cardiovascular disease, neuroprotective, antioxidant, anti-proliferative, neurodegenerative disease
Honokiol	Neolignan biphenols	Inhibition of PI3K/Akt pathway	Anti-thrombotic, anti-inflammatory, anti-oxidant, anti-cancer- multiple myeloma, lung, and colorectal
Matrine	Tetracyclic alkaloid	Inhibition of PI3K/Akt/mTOR pathway	Anti-cancer, anti-inflammatory, anti-viral, anti-fibrotic, anti-arrhythmic, immunosuppressive
Parthenolide	Sesquiterpene lactone	Inhibition of PI3K/Akt pathway	Arthritis, fever, head ache, anticancer- prostate, skin, breast, bile duct, bowel, pancreas
Plumbagin	Naphthoquinone	Inhibition of PI3K/Akt/mTOR pathway	Anti-inflammatory, neuroprotective, hypolipidemic, anticancer, anti-atherosclerotic, anti-fungal, antibacterial
Triptolide	Diterpenoid triepoxide	Inhibition of PI3K/Akt pathway	Arthritis, variety of cancers, anti-inflammatory, immunosuppressive, anti-cystogenesis
Wogonin	Flavone	Inhibition of PI3K/Akt pathway	Anti-bacterial, antiviral, anti-oxidant, anti-inflammatory, anti-cancer
Thymoquinone	Benzoquinone of essential oil	Inhibition of PI3K/Akt pathway	Bronchial headache, asthma, dysentery, gastrointestinal problems
Andrographolide	Labdane diterpenoid	Inhibition of PI3K/Akt pathway	Upper respiratory tract infections, immunomodulatory, anti-cancer
Resveratrol	Natural polyphenol with stilbene	Inhibition of PI3K/Akt pathway	Anti-viral, anti-inflammatory, anti-leukemic, neuroprotective, chemoprotective, anti-oxidant
Epigallocatechin-3-gallate	Polyphenol, catechin, ester of epigallocatechin and gallic acid	Inhibition of PI3K/Akt/mTOR pathway	Cardiovascular disease, anti-cancer, anti-obesity, skin protection from ionizing radiation
Quercetin	Flavonoid	Inhibition of PI3K/Akt pathway	Hay fever, diabetes, peptic ulcer, cataracts, asthma, schizophrenia, gout inflammation, viral infections, anti-cancer
Fistein	Flavonoid	Inhibition of PI3K/Akt/mTOR pathway	Anti-oxidant, anti-inflammatory, anti-cancer
Luteolin	Flavonoid	Inhibition of PI3K/Akt/mTOR pathway	Anti-cancer, anti-oxidant, anti-inflammatory
Apigenin	Flavonoid	Inhibition of PI3K/Akt pathway	Anti-inflammatory, sedative, numerous cancers- prostate, melanoma, colon, and breast
Indole-3-carbinol	Breakdown product of glucosinolate	Inhibition of PI3K/Akt pathway	Numerous cancer types- breast, colon, thyroid, gastric, prostate and mesothelioma
Di-indolylmethane	Derived product from digestion of indole-3-carbinol	Inhibition of PI3K pathway	Anti-cancer
Sulforaphane	Organosulfur compound with isothiocyanate group	Inhibition of PI3K/Akt pathway	Anti-cancer and anti-viral
Phenethyl isothiocyanate	Isothiocyanate derivative	Inhibition of PI3K/Akt/mTOR pathway	Numerous cancers
Benzyl isothiocyanate	Isothiocyanate derivative	Inhibition of PI3K/Akt pathway	Anti-cancer
Dehydroglyasperin D	Prenyl flavonoid	Inhibition of PI3K pathway	Anti-obesity, aldose reductase inhibition, anti-cancer, anti-oxidant
Evodiamine	Quinolone alkaloid	Inhibition of PI3K/Akt pathway	Anti-nociceptive, anti-anoxia and vasorelaxant
Piceatannol	Natural analog of Resveratrol	Inhibition of PI3K pathway	Anti-cancer, anti-inflammatory, atherosclerosis, hypercholesterolemia, angiogenesis
Ellagic acid	Polyphenol	Inhibition of PI3K/Akt/mTOR pathway	Anti-bacterial, anti-viral, anti-cancer

**Figure 2:**
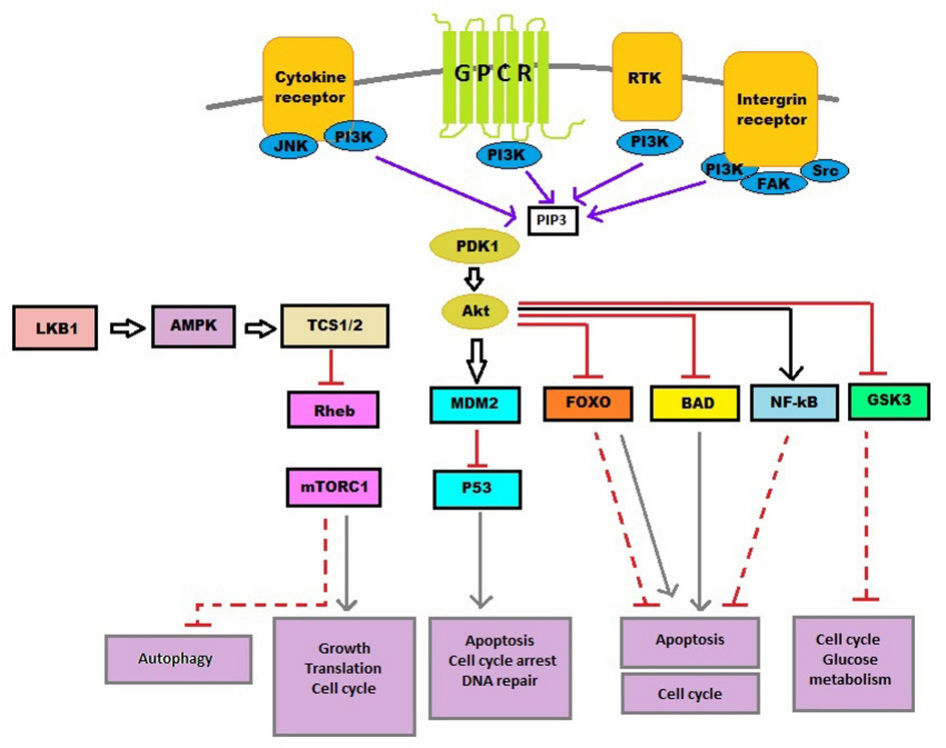
PI3K signaling pathway. Cytokine, growth factor (RKT), hormones and intergrins are prime extra-cellular signals, which tend to transmit via suited pathways to control various cellular processes, like autophagy, DNA repair, cell cycle, angiogenesis, motility, angiogenesis, and senescence. BAD, Bcl-2-associated death promoter; FOXO, Forkhead box protein O; GPCR, G protein coupled receptors; GSK3, Glycogen synthase kinase 3; JNK, c-Jun N-terminal kinases; LKB1, Liver kinase B1; MDM2, Mouse double minute 2 homolog; mTOR C1, Mammalian target of rapamycin complex 1; NF-κB, Nuclear factor kappa-light-chain-enhancer of activated B cells; PDK1, Pyruvate dehydrogenase lipoamide kinase isozyme 1; PI3K, Phosphatidylinositide 3-kinases; PIP3, Phosphatidylinositol (3,4,5)-triphosphate; RHEB, Ras homolog enriched in brain; RTK, Receptor tyrosine kinase; TCS1/2, Two-component signal transduction protein 1/2.

Recently, it is established that many bioactive components modulate cell signaling pathways to mediate the anticancer effect, such as Mitogen-activated protein kinase (MAPK), Wnt and phosphatidylinositol 3-kinase (PI3K)/Akt pathways. This chapter focuses on different phytoconstituents acting on a PI3K signaling pathway that has been responsible for cancer prevention (Reddy et al., 2003). Lipid kinase PI3K exhibits a crucial role in cell cycle, programmed cell death, DNA repair, angiogenesis, autophagy, motility, and cellular metabolism (Cantley, 2002) (Figure 2). PI3K pathway is the most activated route in case of human cancer. Therefore, it is crucial to consider in target specific cancer treatment (Workman et al., 2006; Clarke and Workman, 2012). On activation of PI3Ks, phosphatidylinositol (3,4)-bisphosphate converts to phosphatidylinositol (3,4,5)-triphosphate and engage Akt to cell membrane (Okkenhaug and Vanhaesebroeck, 2003; Yap et al., 2008). Recruitment of Akt leads to conformational change along with Akt phosphorylation followed by its activation. PI3Ks channel signal from cell surface is carried toward cytoplasm by generation of second messengers, phosphorylated phosphatidylinositol which successively phosphorylate downstream substrate and in the end event in proliferation, cell survival, and promote normal cell growth (Cantley, 2002; Osaki et al., 2004; Hu et al., 2012; Akinleye et al., 2013; Burris, 2013). Also PI3K/Akt is found to show cross-talks with other pathways such as MAPK pathway that regulate cell survival or growth. Constitutive activation of PI3K is found in various types of cancers. Aberrant activation of PI3K signaling pathway aid tumor angiogenesis and carcinogenesis (Osaki et al., 2004; Samuels et al., 2004; Slomovitz and Coleman, 2012; Patel, 2013). Other genetic deviations which urge the PI3K pathway in cancer which comprises of gene amplification of PI3Ks, depletion of regulatory activity of PTEN and receptor tyrosine kinase mutation activation. Thus, PI3K is a crucial target for different cancer types and phytochemicals targeting PI3K can be a novel and promising therapy for cancer.

Nucleotide-binding domain, leucine rich repeat containing proteins (NLRs) are intracellular sensors of molecular patterns associated with damage or pathogen entry. It's been reported that NLRs play significant role in regulation of several functional process like inflammation, cell proliferation, cell death, tumourogenesis. Recent study identified that NLRC3 as negative modulator of PI3K-mTOR pathway in tumor suppressor function (Karki et al., 2016). To further investigate the underlying mechanism Rajendra Karki et al. studied on mouse model of colorectal cancer in wild type and NLRC3^+/−−^. Study suggests that NLRC3 negatively regulates the PI3K/mTOR signaling pathway which are crucial for cell proliferation and survival. Still the role of NLRC3 in other pathways to be explored. Further molecular insights of NLRC3 might benefit in the treatment and prevention of metabolic diseases and cancer (Karki et al., 2017).

### Role of PI3K in cancer

PI3K is the potential and druggable target for cancer therapy. Literature suggests that PI3K signaling pathway is activated in almost 30–50% of various human cancers. Based on the structure and specificity of substrates, PI3Ks have been divided into three classes. Most commonly studied are Class I and so far four isoforms of Class 1A PI3K have been identified and encoded as PI3KCA, PI3KCB, PI3KCD, and PI3KCG which catalyzes the phosphorylation of inositol ring at 3′ position. All the isoforms are mediated though GPCRs under the regulatory control of p110α, p110β, and p110γ. Multiple components of the PI3K signaling pathway are activated and mutated in human cancers. Activation of PI3K is linked to increased levels of PIP3 which further activates Akt pathway and leads to cellular progression, survival, and angiogenesis (Liu et al., 2009).

Currently around 50 drugs have been identified targeting PI3K/Akt/mTOR and many of them are under clinical investigation especially of Class 1 pan-PI3K inhibitors. For effective cancer therapy, PI3K inhibitors in combination with pathways targeting Akt, mTOR, RTKs (receptor tyrosine kinases), MAPK (mitogen activated protein kinase), EGFR (epidermal growth factor receptor), HER2 (human EGF receptor 2), DNA repair enzymes (Yap et al., 2015; Massacesi et al., 2016; Pons-Tostivint et al., 2017).

## Gingerols

10-gingerol and 6-gingerol are obtained from Ginger (*Zingiber officinale*), they exhibit anticancer, (Ryu and Chung, 2015) antineuroinflammatory, (Ho et al., 2013) antioxidant, and anti-inflammatory (Dugasani et al., 2010) activities. Zhang et al. assessed the anti-cancer effect of 10-gingerol (10-G) against HeLa cells. The apoptotic effect on cells did not show diverse attribute after 12 h of treatment with 10-G. As the exposure was extended apoptotic cell number was higher in the case of 10-G than that of 5-FU (Zhang et al., 2017). Controlling of PI3K/Akt pathway signal transduction could be linked with the anti-cancer activities of 10-G. Therefore, this head to examine the change of survival pathway related proteins. Former studies suggested that 6-gingerol had no effect on the expressions of PI3K and p85α; but, PI3K regulated an increase in phosphorylation of Akt by 6-Gingerol (Park et al., 2006). The present study investigated 10-G induced apoptosis by PI3K/Akt/AMPK/ mTOR signaling pathways in HeLa cells which were confirmed by significant inhibition of PI3K phosphorylation. Overall results showed that 10-G triggered mTOR-mediated cell apoptosis by inhibiting PI3K/Akt and activating AMPK to induce mTOR-arbitrated cell apoptosis in HeLa cells (Zhang et al., 2017).

Weng et al. studied the molecular mechanism of 6-gingerol and 6-shogaol against hepatocarcinoma cells (HCC). In order to assess the inhibition and the primary molecular mechanism, the transition and translation of matrix metalloproteinases (MMP) and urokinase-type plasminogen activator (uPA), was studied. PI3K pathway inflects MMP expression. Western blots densitometric analyses revealed ≥10 μM 6-Gingerol and ≥2.5 μM 6-shogaol concentrations, notably repressed the phosphorylation of MAPK and PI3K/Akt signaling along with the activation of NF-κB, and translocation of NF-κB and STAT3 (Huynh, 2010). Thus, it was postulated that the target proteins which tend to regulate 6-gingerol and 6-shogaol-mediated inactivation of invasion and metastasis might likewise prevent angiogenesis in HCC (Weng et al., 2012).

## Curcumin

Curcumin (1E,6E)-1,7-bis(4-hydroxy-3-methoxyphenyl)hepta-1,6-diene-3,5-dione is a flavonoid isolated from rhizome of *Curcuma longa* (Minami et al., 2009). It is also used in cardiovascular disease, arthritis, and inflammation, breast cancer, gastric cancer, colon cancer, melanoma, prostate cancer, lymphoma, and leukemia (Shehzad et al., 2013).

Jiao et al. assessed the effect of curcumin in lung cancer, as the possible mechanism of curcumin in epithelial-mesenchymal transition (EMT) and angiogenesis was not known. As EMT and angiogenesis appear as two crucial events in cancer development. HGF/c-Met pathway is important for invasive growth. The involvement of PI3K pathway with activated c-Met intensifies PI3K activity (Eder et al., 2009). In lung cancer other than PI3K pathway, Akt/mTOR signaling pathway also exhibits a crucial role in modulation of EMT, cell growth, cell survival, and cell cycle. In this study, it was revealed that curcumin inhibited HGF induced EMT via inhibition of c-Met activation and PI3K/Akt/mTOR signaling pathway displaying anticancer effect of curcumin (Jiao et al., 2016).

## Diallyl trisulfide

Diallyl trisulfide 3-(prop-2-enyltrisulfanyl) prop-1-ene is a main active compound isolated from Garlic (*Allium sativum*). It is used in diabetes, cardiovascular disease, to stimulate the immune system and against infections. Also, it has potent anticancer property, (Fleischauer et al., 2000; Hsing et al., 2002) induces apoptosis in colon, gastric, prostate, and breast cancer (Chandra-Kuntal and Singh, 2010; Zhang et al., 2015).

Wang et al. (2016) evaluated Diallyl trisulfide (DATS) induced apoptosis in MG63 and MNNG/HOS cell lines. The cells were evaluated with various concentration of DATS for 48 h, whereas in some groups the cell lines were previously treated with N-acetylcysteine (NAC) and then co-treated with DATS. The apoptosis effect was evaluated by western blotting technique. DATS showed an apoptotic behavior due to inactivation of PI3K/Akt pathway, which was reliant on ROS (Reactive Oxygen Species) generation which was highlighted by NAC treatment. Study concluded that antiproliferative and cytotoxic effect in human osteosarcoma cells (HOS) due to a positive correlation between ROS and PI3K/Akt signaling pathway in addition to the mitochondrial event leading to apoptosis. Also, it showed an increase in intracellular ROS level in addition to MG63 and MNNG/HOS G0/G1 phase cell cycle arrest and cell apoptosis which displays a great capability of diallyl trisulfide as an anticancer agent.

## Emodin

Emodin (1,3,8-trihydroxy-6-methylanthraquinone) is an anthraquinone existing in various herbs like *Rheum Officinale, Semen Cassia, Polygonum multiflorum*, and *Polygonum cuspidatum*.

Upregulation of PI3K pathway associated with tumors along with PI3K pathway activation leads to protein kinase B (Akt) phosphorylation (Bartholomeusz and Gonzalez-Angulo, 2012). Akt overexpression is found in many human cancers, where the active Akt promotes resistance to chemo- and radiotherapy (Courtney et al., 2010). Thus, PI3K/Akt pathways inhibition improves response to tumor cells (Yi et al., 2013). Chun-Guang et al. (2010) evaluated the anti-apoptotic effect of emodin on Human Chronic Myelocytic Leukemia K562 Cell Lines. Treatment of these cell lines with emodin showed downregulation of Akt kinase activity. The results suggested that emodin instigated apoptosis via inhibition of PI3K/Akt level, along with upregulation of (Phosphatase and tensin homolog) PTEN.

Cui et al. studied the molecular mechanism of emodin for its chemoprotective activity on HepG2 cells. Emodin treatment instigated apoptosis, in turn resulted in inhibition of PI3K/Akt and ERK (Nakanishi et al., 2005; Cui et al., 2016). Activation of ERK pathway elicited PI3K/Akt pathways activation, possibly by the disappearance of feedback inhibition. Thus, it was concluded Emodin-induced apoptosis resulted in inhibition of PI3K/Akt pathway and activation of p38 mediated through initiation of mitochondrial pathway (Saini et al., 2013).

## Ginsenoside RG3

Ginsenoside Rg3 (2S,3R,4S,5S,6R)-2-[(2R,3R,4S,5S,6R)-4,5-dihydroxy-2-[[(3S, 5R, 8R,9R, 10R,12R,13R,14R,17S)-12-hydroxy-17-[(2S)-2-hydroxy-6-methyl-hept-5-en-2-yl]-4,4, 8,10, 14-pentamethyl-2,3,5,6,7,9,11,12,13,15,16,17-dodecahydro-1H-cyclopenta[a]phenanthren-3-yl]oxy]-6-(hydroxymethyl)oxan-3-yl]oxy-6-(hydroxyl methyl)oxane-3,4,5-triol is obtained from red *Panax ginseng*. The herb possesses anti-proliferative activity against numerous types of cancer such as ovarian, lung and melanoma (Koo et al., 2007; Wang et al., 2007) and 20(s)-ginsenoside Rg3 caused inhibition of varied cancers via inductive effects on several signaling pathways (Iishi et al., 1997; Keum et al., 2003; Lee et al., 2009).

Wang et al. studied the effect of 20(s)-ginsenoside Rg3 on HO-8910 cell lines. These cells were treated with various concentrations of 20(s)-ginsenoside Rg3. Several pieces of evidence suggested that HO-8910 cells propose deregulation of PI3K/Akt pathway, on their treatment with various concentrations of 20(s)-ginsenoside Rg3. (Kar et al., 2012; Wang et al., 2014). It was concluded that treatment with 20(s)-ginsenoside Rg3 resulted in down-regulation of PI3K/Akt pathway, activation of caspase-3 and -9 and inhibition of apoptosis protein (IAP) family proteins which play a significant part in apoptosis.

## D-Rhamnose β-Hederin

D-Rhamnose β-Hederin (DRβH) (3β [(αLarabinopyranosyl) oxy]olean-12-en-28-oic acid) (DRβH), is a triterpenoid saponin isolated from a traditional antitumor Chinese herb, *Clematis ganpiniana* (Ding et al., 2009).

Cheng et al. (2014) evaluated the apoptotic activity of D Rhamnose β-Hederin (DRβH) from *C. ganpiniana* on various breast cancer cells. MCF7 and MDAMB231 were treated with (DRβH). Western blotting technique disclosed suppression in the p-PI3K expression following treatment of MCF 7 and MDAMB231 with DRβH in a time-dependent manner with sustained effect for 48 h (Calleja et al., 2009). Thus, it was concluded DRβH exerted an apoptotic effect through inhibition of PI3K/Akt pathway.

## Honokiol

Honokiol 2-(4-hydroxy-3-prop-2-enylphenyl)-4-prop-2-enylphenol is a phytochemical present in *Magnolia Officinalis* extract. It possesses anti-thrombotic (Zhang et al., 2007), anti-inflammatory (Liou et al., 2003), anti-oxidant effect (Lo et al., 1994) along with anti-cancer activity in multiple myeloma (Battle et al., 2005; Ishitsuka et al., 2005), lung, and colorectal cancer cells (Yang et al., 2002; Wang et al., 2004).

Liu et al. verified the influence of honokiol on various breast cancer cell lines individually in estrogen receptor negative and positive cell lines in addition to cell lines resistant to tamoxifen resistant and Adriamycin resistant. PI3K/Akt/mTOR signaling pathway was shown to lead to anti-estrogen therapy resistance in cell lines of breast cancer (Clark et al., 2002; Normanno et al., 2005; Nahta et al., 2006). Also alterations from G0/1-phase to S-phase of the cell cycle were regulated by mTOR a downstream mediator of PI3K/Akt pathway (Altomare and Testa, 2005). Based on the observations of the study it was hypothesized that greater degree of the reticence of PI3K/Akt/mTOR pathway mediated by mTOR inhibitor intensified the sensitivities of the cells of breast cancer to honokiol. Also the combination treatment of honokiol and mTOR inhibitor would be capable of treatment of breast cancer. Thus, inhibition of PI3K/Akt was predicted be one of the mechanisms for its anticancer activity.

## Matrine

Matrine, is an alkaloid present in *Sophora flavescens Ait*, a Chinese herb. It exhibits several biological activities like anticancer, antiinflammatory, antiviral, antifibrotic, antiarrhythmic, and immunosuppressive effects (Zhang et al., 2001; Long et al., 2004; Li et al., 2009, 2010; Huang et al., 2014; Yong et al., 2015).

Liu et al. evaluated the effect of novel matrine derivative [(6aS, 10S, 11aR, 11bR, 11cS)-10-methylamino-dodecahydro-3a, 7a-diaza-benzo (de) anthracene-8-thione] MASM on human hepatoma cells (HCC) (Hep3B and Huh7) and hepatic cancer stem-like cells (hepatic CSC) Hep3B and Huh7 cell lines. HCC cells were exposed to different concentrations of MASM for 24, 48, and 72 h and hepatic CSC for 24 h, cell proliferation for HCC was evaluated by cell counting kit-8 and programmed cell death by Hoechst 33258 fluorescence staining kit for hepatic SCS respectively. MAMS was found to attenuate phosphorylation of PI3K P110a (a subunit of PI3K) in HCC cells. The PI3K/Akt pathway contributed to, inhibition of MASM on HCC cells and Hepatic CSC. In addition, this study demonstrated that MASM inhibited hepatic CSCs induced cell apoptosis and growth arrest. Further that MASM suppressed hepatoma cell proliferation, and the Akt/mTOR/ p70S6K and Akt/GSK3β/β-catenin signaling pathways, offering possible mechanisms for its antitumor activity (Liu et al., 2016).

## Parthenolide

Parthenolide, is a sesquiterpene lactone isolated from feverfew botanically known as *Tanacetum parthenium*. It is has been preferred in headache, treatment of arthritis and fever (Knight, 1995). Parthenolide also possesses anticancer activity, therefore used against numerous cancers involving organs like bowel, prostate gland, skin, breast, bile duct, pancreas, and others (Zhang et al., 2004; Kim et al., 2005; Liu et al., 2010; Sun et al., 2010; D'Anneo et al., 2013a,b).

Jeyamohan et al. examined the action of Parthenolide on programmed cell death and autophagy in HeLa cells of cervical cancer. HeLa cells lines were treated with different concentrations of Parthenolide and incubated overnight. 3-(4, 5-dimethylthiazol-2-yl)-2, 5-diphenyl terazolium bromide (MTT) assay was carried out to evaluate the cytotoxicity potential of Parthenolide on HeLa cells. The IC_50_ value was found to be 6 μM. p-Akt protein expression was found to be downregulated and ATG3 protein expression was found to be upregulated. It was concluded that Parthenolide resulted in PI3K/Akt pathway inhibition which induces autophagy in Hela cells, triggering apoptosis and autophagy via activation of PTEN expression (Jeyamohan et al., 2016).

## Plumbagin

Plumbagin is a naphthoquinone (PLB, 5-hydroxy-2-methyl-1, 4-naphthoquinone) found in *Plumbago zeylanica* L, *Juglans cinerea, Juglans regia*, and *Juglans nigra*. It is used as anti-inflammatory, neuroprotective agent and it possesses activities like hypolipidemic, anticancer, antiatherosclerotic, antifungal, and antibacterial (Padhye et al., 2012).

Li et al. evaluated the apoptotic action of autophagic cell death in A549 and H23 human non-small cell lung cancer cells. PLB promoted autophagy in A549 and H23 cells through reticence of PI3K/Akt/mTOR pathway by inhibiting the Akt activation along with downstream targets, which includes mTOR, glycogen synthase kinase 3b and forkhead transcription factors (Kuo et al., 2006). It was concluded that PLB inhibited cell proliferation and enhanced intracellular ROS level, predominantly through the activated mitochondria-dependent apoptotic pathway and induced autophagy to a lesser extent in human A549 and H23 cells via PI3K/Akt/mTOR pathway inhibition (Li et al., 2014).

Zhou et al. evaluated the action of Plumbagin (PLB) on human prostate cancer cell lines *viz*., PC-3 and DU145. PLB induced apoptosis was observed and autophagy was analyzed using confocal microscopy and flow cytometric analysis. Cell line incubated with PLB showed an increase in autophagy when incubated for 24 h. It was found that PLB stimulated programmed cell death and autophagy through PI3K/Akt/mTOR-mediated pathway (Zhou et al., 2015).

Wang et al. evaluated the action of PLB in human pancreatic cancer cells which involved PI3K/Akt/mTOR-mediated pathway. PANC-1, and BxPC-3 cells, were exposed to PLB to evaluate the cell killing action. Treatment with PLB on BxPC-3 and PANC-1 cells lead to an evidential change in functional proteins' expression along with its phosphorylation level which modifies autophagy signaling pathway.Thus, a concentration-dependent reduction in the level of phosphorylation was witnessed in the case of PI3K, Akt, and mTOR for the cells treated with different concentrations of PLB. Previous studies revealed the induction of autophagy by PLB in different cancer cell lines via the negative PI3K/Akt/mTOR axis modulation (Kuo et al., 2006; Li et al., 2014) As per Wang's previous data autophagy was induced through inhibition of the PI3K/Akt/mTOR pathway in non-small-cell lung cancer cells. Hence, it was concluded that inhibition of PI3K/Akt/mTOR signaling pathway leads to autophagy effect induced by PLB in PANC-1 and BxPC-3 cells (Sun et al., 2015).

Pan et al. analyzed the effect of PLB on programmed cell death, cell cycle distribution and autophagy, in human tongue squamous cell carcinoma cells. Phosphorylation of phosphatidylinositol, phosphatidylinositol-4, phosphatidylinosi-tol-4-phosphate and 5-bisphosphate catalyzed by PI3K catalyze resulted in the formation of phosphatidylinositol-3, 4, 5-triphosphate. The phosphorylation effect is stimulated by growth factors and hormones, which modulates cell survival, cell cycle migration, and proliferation. In this study, a dose-dependent phosphorylation was significantly inhibited of PI3K at Tyr458 when compared to control. The phosphorylation level of PI3K at Tyr458 was attenuated depending on the concentration of PBL exposed when compared to the control. Thus, it was concluded that PLB inhibited phosphorylation of PI3K at Tyr199 and p38 MAPK at Thr180/Tyr182 but intensify the phosphorylation of GSK3βat Ser9 in SCC25 cells, contributing to the increase in autophagy flux (Pan et al., 2015).

Wu et al. (2016), investigated the potential anticancer effect and mechanism of PLB on multiple myeloma (MM) cells. OPM1 cells were incubated with PBL for 24 h to examine the expression of PI3K and p-Akt using western blot analysis, which disclosed that the anticancer effect of PLB was mediated via the PI3K/Akt signaling pathway in OPM1 cells. Thus, the results of this study concluded, that PLB inhibits cell proliferation and promotes apoptosis of MM cells. Further, the study identified PI3K/Akt/mTOR pathway to be the potential cellular mechanism of PLB in MM cells.

## Triptolide

Triptolide (TPL) is obtained from *Thundergod vine, Tripterygium wilfordii* Hook, and is used as an anti-inflammatory agent in case of rheumatoid arthritis. It is also established for treatment of variety of cancers (Shu et al., 2009; Zhu et al., 2010; Johnson et al., 2011; Manzo et al., 2012; Zhong et al., 2013; Shao et al., 2014; Li H. et al., 2015; Ziaei and Halaby, 2016). However, previous studies have evaluated the anticancer and sensitization effect of TPL in Epithelial Ovarian Cancer (EOC).

Hu et al. evaluated effect of triptolide (TPL) *in vitro* on proliferation, cycle distribution, programmed cell death, and ultrastructure of COC1/DDP cells, also the sensibilization and anticancer effect *in vivo*. The cell lines were exposed to different concentrations of TPL. TPL was found to display apoptosis induction via the restraint of NF-κB in p53-independent pathway, with production of ROS and inactivating the PI3K/Akt signal pathway (Sandler et al., 1997; Lee et al., 2002; Kim et al., 2010; Morgan and Liu, 2011; Zhong et al., 2013; King et al., 2015). The PI3K/Akt pathway tends to upregulate in 30–50% of prostate cancers, and molecular changes have been demonstrated in the PI3K/Akt to distinguish malignant from benign prostatic epithelium and have been related with growing tumor grade, stage, and risk of recurrence (Luo et al., 2003; Hennessy et al., 2005; Morgan et al., 2009). Thus, it was concluded, that TPL boosted cell apoptosis and suppressed tumor growth via the PI3K/Akt pathway. Further that the sound sensibilization effect of TPL assisted DDP to lower the resistance of epithelial ovarian cancer (EOC) to cisplatin (Hu et al., 2016).

Hyoung et al. analyzed, the effect of BIIB021 alone or in combination with the histone acetyltransferase inhibitor (HAT) TPL on the survival of 8505C and TPC-1thyroid carcinoma cells (TCC). The drugs when treated as a single agent shows cytotoxic activities by modulating PI3K/Akt and NF-κB signal pathways (Böll et al., 2009; Xu et al., 2010; Gopalakrishnan et al., 2013; Li et al., 2013; Lin et al., 2014). Thus, they concluded synergistic activity of BIIB021 in combination with TPL participating in suppression of NF-κB and PI3K/Akt/mTOR signal pathways in TCC (Kim et al., 2016).

Miyata et al. demonstrated effect TPL, on human fibrosarcoma in a dose-dependent manner on HT-1080, human squamous carcinoma, and human uterine cervical carcinoma SKG-II cells. TPL initiated inhibition of PI3K which further lead to an increase in JNK1 activation through Akt and/or protein kinase C (PKC)-independent pathway(s). Ras–Raf–MEK1/2 and PI3K–Akt are intracellular signaling pathways which play a crucial role to regulate tumor cell proliferation (Martin, 2003; Osaki et al., 2004), hence the association of a signal pathway with TPL-induced inhibition was examined, using HT-1080 cells. The results revealed the inhibition of the PI3K action of TPL which corresponds to antiproliferative effect in HT-1080 cells via Akt and/or the PKC-independent pathway(s). Also, it was analyzed that evidential reduction in PI3K inhibition by TPL resulted in an activation of JNK1, which was reponsible for its anti-tumor activity. Thus, the evidence that the reduction in PI3K activity by TPL resulted in the inhibition, which further induced apoptosis (Miyata et al., 2005).

## Wogonin

Wogonin, 5,7-dihydroxy-8-methoxy-2-phenylchromen-4-one is the major active constituent present in the root of the *Scutellaria baicalensis* Georgi, a Chinese herb used in various diseases due to its antibacterial, antiviral, antioxidant, anti-inflammatory, and anticancer effects (Li-Weber, 2009; Gasiorowski et al., 2011).

Hu et al. evaluated apoptosis and endoplasmic reticulum stress in HL-60 leukemia cells. HL-60 cells were evaluated with different concentration of wogonin. The viability of HL-60 was inhibited in a dose-dependent manner. The results revealed inhibition of phosphorylation of PI3K by Wogonin at Tyr458 and Akt at Ser473 in concentration dependent manner. It was hypothesized that PI3K/Akt signaling pathway exhibited a crucial role in programmed cell death in HL-60 from previous studies, induction of apoptosis through the PI3K/Akt pathway in HT-29 human colorectal cancer cells (Kim et al., 2012) and in a human myeloma cell line (Zhang et al., 2013). Hence wogonin was proven to possess a potential for human leukemia treatment (Hu et al., 2015).

Huang et al. examined the function of wogonin in the MCF-7 breast cancer cell lines. Western blotting analysis was carried out to study the phosphorylation of PI3K/Akt (p-PI3K/p-Akt). The Western blotting analysis showed decreased in phosphorylation after treatment with wogonin for 24 h and further quenched after 48 h treatment. Thus, it was concluded, that wogonin showed programmed cell death in the MCF-7 breast cancer cell lines, which was linked to down-regulation of survivin and Bcl-2; up-regulation of P53, Bax and caspase-3 activation,. Also, pathways of PI3K/Akt and MAPK/ERK showed significant role in programed cell death of MCF-7 induced by wogonin. Inhibition of the PI3K/Akt pathway by wogonin may be due to down-regulating survivin expression, a downstream target of the PI3K/Akt pathway (Huang et al., 2012).

Further Zhao K et al. observed the activity of wogonin in inhibiting LPS-induced tumor angiogenesis in MCF-7 cells. Western blot investigation was conducted to examine the action of wogonin on PI3K/Akt/NF-κB signaling pathway on cell lines treated with wogonin. Wogonin efficiently suppressed the expression of PI3K and phosphorylation of Akt activated by LPS in a concentration-dependent manner. But the total protein level of Akt remained unaltered. These results suggested that wogonin could block LPS-triggeredPI3K/Akt signaling. Collectively wogonin inhibit LPSþ IGF-1-induced VEGF expression, HUVECs migration, and tube formation via suppression of PI3K/ Akt signaling (Zhao et al., 2014).

## Thymoquinone

Thymoquinone (TQ) 2-methyl-5-propan-2-ylcyclohexa-2, 5-diene-1, 4-dione is a bioactive constituent of black seed oil (*Nigella sativa*). It possesses anti-inflammatory effects and also provides protection against a bronchial headache, asthma, and dysentery, gastrointestinal problems (Woo et al., 2012).

Gemcitabine-based chemotherapy is employed in pancreatic cancer (Stathis and Moore, 2010; Mu et al., 2015). Mu et al. employed a combination treatment of TQ and gemcitabine to aim molecular targets to prevent gemcitabine sensitivity and to induce an apoptotic effect on pancreatic cancer cells, as well as reducing the effective dose of gemcitabine (Banerjee et al., 2009; Rajput et al., 2013). TQ displayed itself as a potential chemosensitizer and apoptotic agent via suppression of the PI3K/Akt/mTOR activation along with suppression of downstream effector S6 ribosomal protein which is associated with the chemoresistance of human malignancies to standard anticancer drugs (Yang et al., 2014). TQ showed chemo-sensitizing and apoptotic effects via a decrease in activation of the downstream effector S6 ribosomal protein and PI3K/Akt/mTOR. Thus, it was concluded that the combination forbids gemcitabine sensitivity and induces apoptosis in pancreatic cancer cells by avoiding Notch1/PTEN, PI3K/Akt/mTOR, NF-κB pathways. Thus, the observation confirmed that the proposed novel synergistic combination leads to reduction in the dose of Gemcitabine which could reduce the risk of gemcitabine- insensitivity and toxicity. This provides a promising approach in human pancreatic cancer treatment.

Iskender et al. evaluated the effects of TQ and myrtucommulone-A (MC-A), EMT on HTB-9 and MDA-MB-231 cell lines. Both MC-A and TQ inhibited epithelial–mesenchymal transition by hindering phosphorylation of several components participating in PI3K/Akt axis as well as MAPK/ERK pathways in a dose dependent manner and reversed TGF-b-induced EMT. MC-A and TQ treatment lead to a consistent decrease in the expression of EMT-related markers. It also obstructed the migratory ability of both HTB-9 and MDA-MB-231 cell lines. The study concluded that MC-A or TQ exhibits antimetastatic effects due to inhibition of PI3K/Akt pathway (Iskender et al., 2016).

Dirican et al. evaluated docetaxel and TQ combination for synergistic cytotoxicity effects and also whether this induction was linked to inhibition of MAPK/ERK and PI3K/Akt pathways. The combination treatment showed inhibition of PI3K/Akt pathway where TQ exhibited a crucial role in synergistic cytotoxic and apoptotic effect in hormone and drug-refractory in DU-145 cell lines. The data revealed a possibility of dose reduction of docetaxel with reduced side effects by its novel combination with TQ which could be of greater potential in patients with castrate-resistant prostate cancer (Dirican et al., 2015).

Xu et al. evaluated the anticancer effect related with PI3K/Akt signaling on TFK-1 and HuCCT-1 cells. *In vitro studies* in cholangiocarcinoma (CCA) cells revealed that TQ controlled PI3K and Akt activation. TQ showed downregulation of p-Akt in both of the CCA cells while Akt protein level left unaltered. In addition, the downregulation of p-Akt was related to the downregulation of XIAP and Bcl-2, as well as the upregulation of BAX, which evidenced apoptosis induction in cells exposed to TQ for 48 h. The results proposed that PI3K/Akt signaling was partially, involved in this effect. TQ mediated inhibition on NF-κB was also tested. The results revealed marked decrease in expression of COX-2, VEGF, and cyclin B1, which tends to be regulated via NF-κB. These observations were invariable with increased inhibition of growth and apoptosis cell death inducing effects, proposing that TQ inhibits DNA-binding activity of NF-κB *in vitro* and the downstream gene products expression. This effect was partially accountable for the increased cell killing effect (Xu et al., 2014). The study also revealed that TQ exhibited a chemopreventive effect against human CCA cells by inhibiting the constitutive activation of proinflammatory transcription factors, including both NF-κB and PI3K/Akt. Thus, it was concluded that TQ treatment results in downregulation of anti-apoptotic and pro-survival proteins which are transcriptionally regulated by the NF-κB and PI3K/Akt pathways, leading to a loss of CCA cell survival and proliferation. Therefore, TQ is stated as a novel therapeutic regimen for the NF-κB and PI3K/Akt pathways inactivation in case of human CCA.

Yu and Kim, examined the effect of TQ on the programmed cell death of chondrocytes on the production of ROS. The chondrocytes were evaluated with increasing concentration of TQ to detect the apoptosis effect. It has been shown that MAPKs and PI3K/Akt signaling pathways could play a role as a mediator in apoptosis (Lee et al., 2000; Aggeli et al., 2006). Also, p38 kinase and PI3K/Akt signaling pathway play an important role in apoptosis induced by TQ. Western blot study detected the expression of PI3K/Akt and MAPKs, p38kinase, ERK-1/-2, and JNKinase, in cells treated with TQ. It resulted in an increase MAPKs and PI3K/Akt, p38kinase, JNKinase and ERK-1/-2 in a dose-dependent manner. Together, the results concluded that TQ lead to ROS induced apoptosis via PI3K/Akt and p38kinase pathways in rabbit's articular chondrocytes. Thus, it was concluded that raised ROS levels tends to inhibit proliferation and cause apoptosis in cells that are malignant by activation of both stress kinase and caspase pathways, which includes MAPKs and PI3K/Akt pathways (Onyango et al., 2005; Zanotto-Filho et al., 2010; Kwon et al., 2011; Singh et al., 2011). It was found that ROS induced by TQ enhanced the PI3K/Akt activation and MAPKs, which also leads to apoptosis. Thus, TQ is capable not only to inhibit proliferation but also to induce apoptosis through p38kinase and Akt pathways (Yu and Kim, 2013).

## Andrographolide

Andrographolide,(3E,4S)-3-[2-[(1R,4aS,5R,6R,8aS)-6-hydroxy-5-(hydroxymethyl)-5,8a-dim-ethyl-2-methylidene-3,4,4a,6,7,8-hexahydro-1H-naphthalen-1-yl]ethylidene]-4-hydroxyoxolan-2-one is the bioactive constituent present in the *Andrographis paniculata* extract. It is also utilized for treating upper respiratory tract infections, fever, diarrhea, rheumatoid arthritis, and recently, also shown to possess anti-inflammatory, immune modulatory, anticancer effects (Li et al., 2007)

Kumar et al. studied andrographolide induced programmed cell death and autophagy in U937 cells, human leukemic cells. U937 cells were treated with andrographolide and further densitometric analysis was conducted. As pathway of PI3K/Akt/mTOR has prototypic functions in cellular proliferation, growth, differentiation, and survival, inhibition of these pathways would be a promising tool against cancer (LoPiccolo et al., 2008). The phosphorylated PI3K expression (pPI3K) was markedly attenuated in AG−4 treated cells in case of both in phosphorylated forms of 85 and 55 PI3K compared to control, in a time-dependent way. Densitometric analysis disclosed the ratios of pPI3K (p85)/total PI3K, time-dependent drop-off after AG−4 treatment by 78.9–97.8% as against the control cells. Thus, the downregulation of PI3K/Akt tends to have a crucial role in AG−4 induced cytotoxicity. Hence it was concluded that AG−4 regulates pPI3K, pAkt, pmTOR, along with other key molecules pPDK1, pcRaf and pGSK3β of PI3K/Akt/mTOR pathway. Further examination demonstrated that AG−4 elicited cytotoxicity which involved redox imbalance and apoptosis cell death by inducing mitochondrial depolarization and the caspase cascade activation related to inhibition of pathway of PI3K/Akt/mTOR (Kumar et al., 2015).

Li et al. studied inhibition of HIF1 by andrographolide through PI3K/Akt pathway. PI3K/Akt signaling downregulation by Andrographolide prevents invasion and migration of A549 cells human non–small cell lung cancer (Lee et al., 2010). Previous data suggested a translation of HIF1α mRNA is under the criterion of a PI3K signaling pathway in several cell types (Laughner et al., 2001; Treins et al., 2002). To analyse the effect of Andrographolide on HIF1α upstream PI3K/Akt/mTOR pathway, experiments were carried out on MDAMB231 cells, which revealed phosphorylation under hypoxia on mTOR, 4EBP1, and P70S6K. The results revealed attenuation in the Akt, mTOR, and p70s6k phosphorylation level along with suppressed HIF1α activity by inhibiting the upstream pathway of PI3K/Akt/mTOR. The PI3K/Akt pathway plays a crucial role in the controlling of HIF1α translation and synthesis in certain types of cancer cells (Jiang and Liu, 2008; Li et al., 2015). These observations supported the possibility that Andrographolide mediates HIF1α inhibition, which might be dependent on PI3K/Akt pathway by preventing HIF1α translation (Li et al., 2007).

Lee et al. also studied the inhibitory effects of andrographolide on migration and invasion in cells lines (A549) of human non-small cell lung cancer. A549 cell lines were incubated with andrographolide for 24 h. As per previous data, MAPK and PI3K/Akt is associated with NSCLC cell metastasis and migration (Choudhury et al., 1997; Duan et al., 2000). The incubated cells showed a concentration-dependent decrease of phospho-Akt and PI3K levels and no effect on MAPK including p38MAPK, N-terminal kinase (JNK), c-Jun and an extracellular signal-regulating kinase 2 (ERK2). Depending on the dose or concentration range, andrographolide restrained the PI3K expression and phospho-Akt coexisting with cell invasion and migration mechanism. Thus, it was concluded Andrographolide treatment prevented cell migration/invasion through PI3K/Akt signaling down-regulation and a further c-Jun/c-Fos (heterodimer complex AP-1) inactivation, that was followed by a depletion of expression MMP-7 (Lee et al., 2010).

Li et al. further analyzed the inhibitory growth effect and mechanisms of andrographolide on U251 and U87 cell lines, human glioblastoma cells. As per studies deactivation of PI3K/Akt signaling has been said as an important target glioblastomas treatment (Kubota et al., 2000; Gharbi et al., 2007). Andrographolide prevented the invasion and migration in cell lines A549 via PI3K/Akt signaling down-regulation (Lee et al., 2010) which plays an essential role in andrographolide-induced prohibition of proliferation and G2/M arrest. Yanchun Li observed that andrographolide exposure markedly attenuated the phosphorylated levels of PI3K, Akt, mTOR, and p70s6k proteins in both U251 and U87 cells. Also, andrographolide effect on signaling of PI3K/Akt prevented G2/M arrest and proliferation in glioblastoma cells, U251 and U87 cells when evaluated in combination with PI3K/Akt inhibitor LY294002. It showed an additive effect on G2/M arrest and proliferation inhibition. Thus, it was concluded that andrographolide inactivated the prosurvival signaling of PI3K/Akt, which may play a critical role in the G2/M arrest and prevents proliferation by andrographolide in human glioblastoma cells (Li et al., 2012).

## Resveratrol

Resveratrol (5-[(E)-2-(4-hydroxyphenyl) ethenyl] benzene-1, 3-diol) is obtained from red wine, peanuts, grapes. It has antiviral, anti-inflammatory, antileukemic, neuroprotective, chemoprotective, and anti-oxidant activities. It is effective as an anticancer agent.

As per Jiang and Liu, PI3K/Akt/mTOR pathway was downregulated by resveratrol. Induction of cell death by inducing caspase-3 activation carried out by signaling proteins was found to be enhanced by resveratrol (Jiang and Liu, 2008; Jing et al., 2016).

Rachid et al. investigated mechanism of resveratrol mediated regulation of the gene expression of the genes that are a part of PI3K/Akt signaling in cells of human breast cancer (MDA-MB-231). They used a microarray that is PI3K-Akt pathway-focused with 96 genes, associated with PI3K-Akt signaling pathways. For signal normalization, they used 10 house keeping genes. As per their results, PDPK1 was the gene in the microarray which was upregulated by more than 15-fold. This is the kinase that phosphorylates RSK, PKB/Akt. According to their data, after resveratrol treatment, there was an alteration in 13 genes. Also, c-fos and P70S6K were regulated (Rachid and Alkhalaf, 2006).

Kwon et al. studied the apoptosis induction in cells of breast cancer (MDA-MB-231) by resveratrol. Their result of MTT assay showed that there were a time and concentration dependent reduction in viability of the cell. As per their results, there was a time-dependent reduction in the expression of PI3K/Akt by resveratrol. On the other hand, the expression of cleaved-caspase-3, p53, and cleaved-caspase-9, was increased. Tumor volume was significantly decreased (Kwon J. K. et al., 2012).

As per Frojdo et al. (2007) resveratrol inhibited the catalytic subunits of PI3K class IA *viz*. p110α and p110β which further blocks PI3K-PKB pathway. Resveratrol also inhibits the phosphorylation of PI3-kinase (Frojdo et al., 2007). Immunoprecipitation assay and kinase assay by Benitez et al. in LNCaP and PC-3, resveratrol led to inhibition of Era- and AR-dependent PI3K activities. This led to decrease in protein kinase B/Akt phosphorylation and also the phosphorylation of glycogen synthase kinase-3 (GSK-3). The dephosphorylation of GSK-3 further led to decrease in cyclin D1 levels (Benitez et al., 2007).

Wang et al. investigated the resveratrol utility in PC-3 cancer cells. The purpose was to study its effect on apoptosis of prostate cancer and EMT. For this, they used Western blotting using real-time PCR, and fluorescence-activated cell sorting etc. As per their results, resveratrol modulates EMT and apoptosis through PI3K/Akt pathway in prostate cancer (Wang et al., 2016). As per studies carried out by Aziz et al. resveratrol inhibited the activation of PI3K/Akt. This lead to apoptosis of LNCaP cells by modulating Bcl-2 family proteins. Thus, resveratrol was found to be effective in prostate cancer by inhibiting PI3K (Aziz et al., 2006).

## Epigallocatechin-3-gallate

Epigallocatechin-3-gallate [(2R,3R)-5,7-dihydroxy-2-(3,4,5-trihydroxyphenyl)-3,4-dihydro- 2H-chromen-3-yl] 3,4,5-trihydroxybenzoate (EGCG) is the main catechin extracted from green tea. It accounts for about 50–80%. It is used in improving cardiovascular health, in cancer chemoprevention, it protects skin from damage caused by ionizing radiation, and it is used as the anti-obesity agent.

The effect of EGCG in pancreatic cells on PI3K/Akt/mTOR pathway was determined by Liu et al. They utilized MTT assay to study the cell proliferation and flow cytometry was utilized to study apoptosis of PANC-1 cells. They utilized western blotting to evaluate the expression of proteins involved while RT-PCR was utilized to determine the expression of the genes which are a part of PI3K/Akt/mTOR pathway. Their observations indicated that the proliferation of PANC-1 cells was inhibited by EGCG and it also induced apoptosis. It upregulated protein and PTEN mRNA expression levels and downregulated the expression of phosphor-mTOR and phosphor-Akt (Liu et al., 2013).

Nomura et al. studied the effect of EGCG PI3K (activated UV-B) in epidermal cells JB6 Cl 41 of a mouse. As per their results, the activation of PI3K via UVB was inhibited by pretreatment of these cell with EGCG. Further, activation of PI3K and Erk in UVB signaling was inhibited by EGCG. Also, it attenuated activation of p70 S6-K and Akt which are downstream effectors of PI3K (Nomura et al., 2001).

## Quercetin

Quercetin [5,7-dihydroxy-2-(4-hydroxyphenyl)chromen-4-one] is obtained from green tea, red wine, apples, onions, *Ginkgo biloba*, St. John's wort. Buckwheat tea contains a major amount of quercetin. It is used for treatment of hay fever, diabetes, peptic ulcer, cataracts, asthma, schizophrenia, gout inflammation, viral infections, and cancer.

Xiang et al. explored how quercetin affects the proliferation and apoptosis of HeLa cells. They incubated HeLa cells with quercetin at various concentration levels. They determined the cell viability by MTT assay, cell apoptosis with Annexin-V/PI double labeled cytometry and DNA ladder assay. Cell cycle was flow cytometrically determined. They used a fluorescence microscope after Hoechst 33258 staining to observe the changes in the morphology in the cells. Western blotting was performed to evaluate the proteins related to apoptosis in the HeLa cells. Their results showed that quercetin caused inhibition of HeLa cell growth and induced apoptosis *in vitro* in a time- and concentration-dependent manner. As per their results, quercetin treatment led to the downregulation of the PI3K and p-Akt expression. In addition, quercetin could down-regulate expression of bcl-2, up-regulate Bax, but exerted no effect on the overall expression of Akt. Thus, they concluded that quercetin induced apoptosis via PI3K/Akt pathways (Xiang et al., 2014).

Study of Maurya et al. used Dalton's lymphoma mice to study the mechanism of quercetin regulating PI3K/Akt pathway. They analyzed the effect of quercetin on the expression and the levels of Akt1, p53, and PI3K in ascite cells. As per their results, in ascite cells of Dalton's lymphoma mice, PI3K signaling was hyperactivated. This led to Akt activation and p53 inactivation. Also, the longevity of mice and morphological parameters confirmed tumor suppressor activity of quercetin. Thus, it was concluded that quercetin may lead to prevention of lymphoma prevention by downregulation PI3K–Akt1–p53 pathway (Maurya and Vinayak, 2016).

## Fistein

Fistein [2-(3, 4-dihydroxyphenyl)-3, 7-dihydroxychromen-4-one] is obtained from vegetables and fruits such as grape, strawberry, cucumber, and persimmon. It possesses antioxidant, anti-inflammatory, anticancer and scavenging activities (Khan et al., 2013).

Adhami et al. studied effect of fistein against prostate, pancreatic, and lung cancer. For their study, they used prostate and lung adenocarcinoma cell lines. According to their results, fistein inhibited PI3K/Akt pathway and mTOR pathway. Also fistein when used in combination with other anticancer agents, enhanced their cytotoxic effects (Adhami et al., 2012).

Khan et al. found out that in NSCLC cells fistein suppressed the signaling of PI3K/Akt and mTOR and also inhibited cell growth. Fistein inhibited phosphorylation of mTOR, Akt, eIF-4E, p70S6K1, and 4E-BP1. PI3K (p85 and p110) protein expression was also reduced by fistein (Khan et al., 2012).

## Luteolin

Luteolin [2-(3,4-dihydroxyphenyl)-5,7-dihydroxychromen-4-one] is distributed in a many of vegetables, fruits such as celery, olive oil, peppers, rosemary, peppermint, oregano, and thyme. Luteolin has anticancer, antitumorigenic, antioxidant, radical scavenging, and anti-inflammatory activities.

Hong et al. (2014) described luteolin mediated inhibition of the growth of the NSCLC tumor in mice and T790M mutant NSCLC cells proliferation. According to their observations, luteolin inhibited the binding between mutant EGF receptors and Hsp90 and thus led to degradation of EGF receptors. PI3K/Akt/mTOR pathway for cell survival, cell proliferation Ras/Raf/MAPK pathway are the two main downstream signaling pathways of EGF receptors. In this study, luteolin caused degradation of mutant EGF receptors. It mainly affected PI3K/Akt/mTOR pathway causing a reduction in its activity.

As per the Western blot analysis and kinase assay carried out by Kim et al. (2013). luteolin inhibited expressions of PI3K and Raf and it also attenuated the Akt and MEK phosphorylation. They also carried out a pull-down assay. As per the results of this pull-down assay, there was a non-competitive binding of luteolin with ATP which suppressed Raf activity whereas there was competitive binding of luteolin to ATP which resulted in inhibition of PI3K activity. There was inhibition of tumor volume and tumor nodules by luteolin as shown by an *in vivo* mouse study (Hong et al., 2014).

## Apigenin

Apigenin [2-(3, 4-dihydroxyphenyl)-5, 7-dihydroxychromen-4-one] is mainly found in onions, tea, parsley, and wheat sprouts. It exhibits therapeutic activities such as anti-inflammatory, sedative and it is also used in prevention and treatment of prostate cancer. It suppresses angiogenesis and tumorigenesis in melanoma, skin carcinoma, colon carcinoma, and breast carcinoma.

Ruela-de-Sousa et al. studied the action of apigenin on two models of leukemia including erythroid (TF1 cells) and myeloid (HL60 cells). In these two models, they induced cell death and cell-cycle arrest. According to the observations, apigenin activated PTEN and downregulated PI3K and PDK-1 which led to inhibition of PKB/PI3K pathway in HL60 cell lines. However, under apigenin treatment, TF1 did not show any change in PI3K/PKB pathway (Ruela-de-Sousa et al., 2010).

Erdogan et al. analyzed the effect of apigenin on apoptosis, cell stemness, survival, and migration properties in CSCs. Since PI3K/Akt signaling regulates PCa proliferation and survival, and the inhibition of Akt phosphorylation (pAkt) downregulates NF-κB activation; they decided to analyze the effects of apigenin on this signaling pathway. It was demonstrated that PI3K and NF-κB were predominantly activated and Akt was predominantly phosphorylated in control CSCs. Treating the cells with apigenin markedly inhibited the expression levels of Akt, PI3K, and NF-κB p105/p50 proteins, and inhibited the phosphorylation of pAkt (Erdogan et al., 2016).

Protein kinases play a crucial role in signal transduction pathways that regulate proliferation of cells, their differentiation, and apoptosis. Therefore, agents which could regulate cellular growth by modulating kinases may be developed as an effective anticancer agent. Shukla et al. evaluated the effect of apigenin in cancer cells of the human prostate. As per their results treatment of asynchronized androgen-responsive LNCaP and cell lines of androgen-refractory PC-3 with apigenin (1–40 μM) led to the enhanced arrest of the G0-G1 phase cells of the cell cycle. Further treatment of cells with apigenin caused a time and dose-dependent decrease in both retinoblastoma (Rb) protein phosphorylation at Ser807/811 and Ser780 total Rb protein. Apigenin led to MAPK pathway activation and decrease in expression of cyclin D1 protein which in turn decreased the phosphorylation and protein expression of PI3K-Akt and p38. In addition, apigenin led to the loss of phosphorylation of RNA polymerase II, thus inhibiting transcription of the proteins (Shukla and Gupta, 2007).

Way et al. examined the effect of apigenin on many cell lines of human breast cancer exhibiting various levels of HER2/*neu* expression. They found that apigenin had potent growth inhibitory activity on these cell lines. But in cells with basal levels of HER2/neu, apigenin was less effective. They also investigated the role of PI3K and Akt in cell survival pathway. According to the results, apigenin directly inhibited PI3K activity while the activity of Akt kinase was indirectly inhibited. They also observed inhibition of HER2/*neu* autophosphorylation and transphosphorylation. Further apigenin prevented the PI3K docking to HER2/HER3 heterodimers and thus inhibited the activity of Akt kinase. Thus, they concluded that cellular effects of apigenin resulted from the loss of expression of HER3 and HER2/neu and inactivation of PI3K and Akt (Way et al., 2004).

## Indole-3-carbinol

Indole-3-carbinol [1H-indol-3-ylmethanol] (I3C) is found in cruciferous vegetables such as Brussels cabbage, sprouts, broccoli, cauliflower (Pfeifer and Fahrendorf, 2015). It is mainly used in breast, colon, thyroid, gastric, prostate cancer, and mesothelioma (Fares, 2014).

Mao et al. investigated inhibition of NPC cells by I3C. Various concentrations of I3C were applied on NPC cell lines. The cell proliferation was analyzed at certain time intervals (0, 24, 48, and 72 h). After 48 h, the PI3K/Akt associated proteins levels were determined. Tumors were induced in nude mice and the PI3K/Akt pathway associated proteins levels were determined. According to their results, as the concentration of I3C increased, in PI3K/Akt pathway associated proteins levels and cell proliferation was decreased while there was an increase in apoptosis. Also in the prevention and treatment groups of mice, there was the development of small tumors and also the levels of PI3K/Akt pathway proteins were reduced (Mao et al., 2014).

## Di-indolylmethane

Di-indolylmethane [3-(1H-indol-3-ylmethyl)-1H-indole] (DIM) is obtained from condensation reaction of I3C. I3C is used in cancer treatment, it has the disadvantage of being highly unstable. I3C molecules combine in the acidic pH of the stomach and lead to the formation of a complex mixture of therapeutically active compounds. From this mixture of compounds the most prominent acid condensation product of I3C is the dimer DIM. The yield of DIM is only 10–20% of the products formed. Formation of DIM from I3C is the pre-requirement for the anti-cancer activity of I3C as it the most potent condensation product (Ahmad et al., 2013). It is used to prevent uterine cancer, cancer, and colorectal cancer. It is utilized in the treatment of premenstrual syndrome and it prevents an enlarged prostate (benign prostatic hypertrophy, BPH).

Qian et al. assessed the effect of DIM given intranasally against lung tumorigenesis. Lung tumorigenesis was induced in mice using 4-(methylnitrosamino)-1-(3-pyridyl)-1-butanone (NNK). According to their observations, after administration of DIM, there was 72% reduction in lung tumor multiplicity. In the case of large tumors, there was complete abolishment and for 0.5–1 mm tumors there was 74% reduction. There was 82% reduction in tumor volume. Further they used *in vitro* lung tumorigenesis model for further studies which showed that in both premalignant and malignant bronchial cells, DIM showed apoptotic and antiproliferative effects and in parental immortalized cells there were minimal effects. These effects were found to be, at least in part through PI3K pathway suppression (Qian et al., 2013).

## Sulforaphane

Sulforaphane [(E)-4-isothiocyanato-1-methylsulfinylbut-1-ene] (SFN) is found in cruciferous vegetables and is a major constituent of radish seeds. It shows antimutagenicity, anticancer, and antiviral activities (Yang et al., 2016).

Effect of SFN on four breast cancer cell lines showed abnormalities in ErbB2/ER-PI3K-Akt-mTOR-S6K1 signaling pathway as reported by Pawlik et al. The breast cancer cell lines used include, MDA MB 468, MDA MB 231, SKBR3, and MCF-7. They determined Akt phosphorylation status, cell viability, protein synthesis, cell ultrastructure, autophagy induction, and S6K1 kinases status following SFN treatment. According to their results, all four cell lines showed similar sensitivity to SFN mediated via inhibition of PI3K (Pawlik et al., 2013).

Influence of SFN on ovarian cell lines growth *viz*; SKOV-3, OVCAR-3, and MDAH 2774 were carried out by Chuang et al. The pathway that was influenced was determined by Chaudhuri et al. while the signaling mechanism by which SFN influences the ovarian cancer cell proliferation and growth was determined by Bryant et al. According to Chuang et al. there was a concentration dependent decrease in cell density. They also carried out analysis of the cell cycle phase progression which showed that in G2M and S phases there was a decrease in cell population while there was an increase in G1 phase cell population which showed arrest of G1 cell cycle. The decrease was found to be dependent on time and concentration. These results indicated the role of SFN in causing apoptosis and growth arrest in cell lines of ovarian carcinoma. Chaudhuri et al. studied the effect of SFN on Akt signal transduction pathway. According to their results, in cell lines of ovarian cancer exposed to sulforaphane, there was a reduction in PI3K, active phosphorylated levels of Akt and total Akt protein. Bryant et al. used gene expression profile analysis and found out that SFN causes the arrest of G1 cell cycle (Chuang et al., 2013).

## Phenethyl isothiocyanate

Phenethyl isothiocyanate [2-isothiocyanatoethylbenzene] (PEITC) is mainly found in crucifers such as cauliflowers and olives. It is mainly used in the treatment of various types of carcinomas.

Li et al. analyzed the effect of phenethyl isothiocyanate (PEITC) on metastasis and invasion of colon cancer. They cultured SW480 cells for 24 h with PEITC. MTT assay was utilized to determine cell proliferation. For determination of cell invasion, wound healing assay was used while for determination of cell migration transwell invasion assay was performed. The expression of MMP-9 mRNA was determined using qRT-PCR. The Western blotting study was done to analyze the PI3K and PTEN expression, mTOR and Akt phosphorylation and nuclear translocation of nuclear factor-kappa B (NF-κB). PEITC inhibited invasion and migration of SW480 cell within toxicity-free dose ranges. According to western blotting and molecular data, the expression of PI3K and phosphorylation of mTOR and Akt was inhibited by PEICT (Li et al., 2016).

## Benzyl isothiocyanate

Benzyl Isothiocyanate [isothiocyanatomethylbenzene] (BITC) is obtained from cruciferous vegetables such as garden cress. It is mainly used in cancer treatment.

Boreddy et al. hypothesized that the pathway PI3K/Akt/FOXO may be associated BITC induced apoptosis. According to their results, BITC caused tumor growth suppression and enhanced tumor cell apoptosis. This was related to inhibition of PI3K, Akt and FOXO activation. Using Western blot analysis, immunofluorescence, kinase activity, and EMSA it was confirmed that the activated levels of FOXO, PI3K, and Akt were inhibited in pancreatic cells including PanC-1 and BxPC-3. BITC reduced phosphorylation rather than protein levels of PI3K in BITC-treated tumors and both PanC-1 and BxPC-3 cell lines. Thus, it was concluded that BITC targeted activated-PI3K and not protein levels to inhibit the growth of pancreatic cancer cells (Boreddy et al., 2011).

## Dehydroglyasperin D

Dehydroglyasperin D (DHGA-D) is mainly found in liquorice. It has activities such as antiobesity, aldose reductase inhibitory activities, anticancer, and antioxidant activities.

The action of DHGA-D on proliferation of colorectal cancer cells was evaluated by Jung et al. They also determined that primarily DHGA-D targets signaling. In HT-29 human colorectal adenocarcinoma cells, they studied anchorage-independent and anchorage-independent cell growth. They used Western blot analysis along with a particular antibody to determine the target protein of DHGA-D. Kinase and pull-down assays were used to determine the direct interaction between the target protein and DHGA-D. Further to determine the signaling pathway, they used techniques such as flow cytometry and western blot analysis. Pull-down assays and kinase assays showed that PI3K activity was directly suppressed by DHGA-D. According to their study PI3K inhibition downregulated cyclin D1 expression and GSK3β and phosphorylation thus lead to G1 cell arrest in melanoma (Jung and Jeong, 2016).

## Evodiamine

Evodiamine is a quinolone alkaloid obtained from a traditional Chinese medicinal plant, Wuzhuyu (*Fructus Evodiae Rutaecarpae*). Evodiamine exhibits pharmacological activities such as antinociceptive, anti-anoxia, and vasorelaxant properties (Lijuan et al., 2016).

The effects of evodiamine and its mechanism with respect to ovarian cancer cells were explored by Lijuan et al. They treated human ovarian cancer cells (HO-8910PM) with evodiamine and used MTT assay to study the growth inhibition. Propidium iodide (Annexin V-FITC/PI) double staining/Annexin V-fluorescein isothiocyanate assay was used to assess apoptosis induction. Western blot analysis was used to determine the mechanism of apoptosis. Also, the expression levels of proteins of PI3K/protein kinase B (Akt) pathway and/or MAPK were determined. According to their results, evodiamine inhibited the HO-8910PM cells proliferation in a concentration and time-dependent manner. Evodiamine decreased activity and expression of Akt, PI3K, p38 MAPK and extracellular ERK1/2 MAPK (Lijuan et al., 2016).

Evodiamine effect on K1 cell line of papillary thyroid cancer and its mechanism of action were investigated by Lv et al. MTT assay was used to analyze the action of evodiamine on the proliferation of K1 cells. Western blot analysis was used in order to evaluate the expressions of proteins related to apoptosis. According to their results, evodiamine inhibited K1 cells proliferation in a concentration-dependent manner. Furthermore, evodiamine treatment markedly increased ROS generation and LDH leakage. Also, evodiamine downregulated the expression of p-Akt and PI3K which confirmed association of PI3K/Akt signaling pathway with apoptosis induced by evodiamine (Lv et al., 2016).

## Piceatannol

Piceatannol {4-[(E)-2-(3, 5-dihydroxyphenyl) ethenyl] benzene-1, 2-diol} is resveratrol's natural analog present in red wine, peanuts, grapes (Ko et al., 2012).

It exhibits anticancer and anti-inflammatory properties. It is also used in atherosclerosis, hypercholesterolemia, and angiogenesis (Kershaw and Kim, 2017).

Ko et al. used MDA-MB-231 cells to study the mechanisms anti-invasion shown by piceatannol. According to their result, piceatannol caused a reduction in serum-induced cell invasion, adhesion, and migration but viability of cells was not affected. Further, it inhibited protein levels and mRNA expression, matrix metalloproteinase-9 (MMP-9) activity. It increased tensin homolog (PTEN) and phosphatase and reduced phosphorylation of Akt and phosphoinisitide-3-kinase (PI3K). It also inhibited DNA binding of NF-κB on MMP-9 promoter and nuclear factor kappa B (NF-κB) transcriptional activity (Ko et al., 2012).

The role of piceatannol in adipogenesis and its mechanism was determined by Kwon et al. According to their observations piceatannol supressed 3T3-L1 preadipocytes adipogenesis at non-cytotoxic concentrations. It also revealed that activity of PI3K and IR kinase is inhibited by piceatannol (Kwon J. Y. et al., 2012).

According to Song et al. piceatannol inhibited invasive phenotype of MCF10A human breast epithelial cells harboring mutated H-ras (H-ras MCF10A cells) and MMP-2 induced by H-ras more efficiently as compared to resveratrol. According to their results, piceatannol reduced the H-ras-induced phosphorylation of Akt in a time- and concentration-dependent manner. *In vitro* kinase assays revealed that, the activity of PI3K and expression of phosphatidylinositol (3, 4, 5)-trisphosphate (PIP3) in the H-ras MCF10A cells was suppressed by piceatannol. *Ex vivo* pull-down assays showed that there was direct binding of piceatannol to PI3K and thus inhibited its activity (Song et al., 2013).

## Ellagic acid

Ellagic acid {2,3,7,8-Tetrahydroxy-chromeno[5,4,3-cde]chromene-5,10-dione} is found in strawberries, blackberries, walnuts, and raspberries. It is used to treat bacterial and viral infections and to prevent cancer.

Kowshika et al. used hamster model to examine the expression of protein and transcription of VEGF, hypoxia-inducible factor-1alpha (HIF-1a), members of MAPK, and PI3K/Akt signaling pathway. To determine the mechanism by which ellagic acid works they carried out cell culture experiments and molecular docking studies using endothelial cell line ECV304. They found out that ellagic acid downregulates PDK-1, PI3K, mTOR, p-JNK, p-Aktser473, and p-ERK and thus inhibit HIF-1a-induced VEGF/VEGFR2. It also inhibited hypoxia-induced angiogenesis and neovascularization that reduced the expression of histone deacetylase. Ellagic acid suppressed HDAC-6 in ECV304 cells. Molecular docking studies showed that an interaction between ellagic acid and upstream kinase was responsible for regulation of angiogenic signaling (Kowshik et al., 2014).

## Tiebtan medicine

Tang-Kang-Fu-San (TFKS), a traditional herbal formulation prepared by following the principles of textbook of Tibetan medicine. It is formulated with 11 herbs and is being widely used in different parts of China for the treatment of type 2 diabetes. Formulation also has lot of scientific evidence and clinically proven. To further understand the chemical composition and mechanism, Bailu et al. carried out HPLC fingerprint analysis and in vivo mechanistic studies. Fingerprint analysis could mark 13 peaks and identified one of the chief constituent as gallic acid. Under *in vivo* mechastic studies, herb was observed for impaired insulin tolerance in db/db mice and study identified improved glucose tolerance in mice treated with this Tibetan medicine. To further understand the molecular mechanism, western blotting assay was carried out to measure the level of protein expression of PI3K/Akt and AMPK pathways. And found that NLRC3 mRNA levels were significantly increased compared to metformin which can supresses the downstream of PI3K/Akt pathway to modulate the mTOR activity. The present study outlines that TFKS might be associated with genes regulating PPARγ and NLRC3 or by effecting the potential signal factors of PI3K/Akt or AMPK pathways (Bailu et al., 2017).

## Conclusion

Phytochemicals are the paramount cost-effective treatment option for the cancer patients. They are believed to play a crucial role in inhibiting, controlling or blocking the signals that lead to the conversion of normal cells to cancerous cells. Phytochemicals are widely investigated for their potential in cancer treatment and some of them are effective in preventing and treating cancer.

PI3K pathway has a major role in the regulation of growth, proliferation, and survival of cells. Hyperactivation of the PI3K is observed in many types of cancers. It is also implicated in resistance to anticancer treatments such as tyrosine kinase inhibitors, biologics, and radiation. In therapeutic sensitivity restoration, inhibitors of the PI3K are tested in combination with the other anticancer treatments in preclinical and clinical studies. The literature has reported various phytochemicals that have shown effective anticancer activity by targeting PI3K. Table 2 provides a detailed overview of clinical trials reported in literature.

**Table 2:** Clinical trials of phytochemicals for treatment of cancer.

**Molecule**	**Source**	**Clinical trial**	**Targeted population**	**Status**	**Highlights**	**Reference**
Curcumin	*Curcuma longa* (Zingiberaceae)	Colon cancer	18 years and, both sex	Terminated	A randomized, open label clinical trial was carried out to evaluate the effect on abnormalities in colon lining which might tend to adenocarcinoma Dose: 250 mg BID	http://clinicaltrials.gov/show/NCT00176618
		In the lower gastrointestinal tract in Colon cancer	18–85 years, both sex	Ongoing (Phase II)	A non-randomized, crossover, open labeled clinical trial was carried out on adenomatous polyps in colon cancer patients	http://clinicaltrials.gov/show/NCT00248053
		Curcumin in colon cancer for its Chemopreventive effect	18 years and older, both sex	Ongoing (Phase II)	Randomized, Double Blind Placebo-Controlled Trial of Curcuminoids. Action of COX-2 Expression and Cellular Proliferation, Apoptosis in the Colorectal Mucosa of patients with currently Resected Sporadic Adenomatous Polyps Dose: 4 g	http://clinicaltrials.gov/show/NCT00118989
		Curcumin in smokers with aberrant crypt foci in case of colon cancer	40 years and older, both sex	Ongoing (Phase II)	Non-randomized open label trial to study how well curcumin works in controlling occurrence of colon cancer in smokers having aberrant crypt foci Dose: 2g, 4g.	http://clinicaltrials.gov/show/NCT00365209
		To treat familial adenomatous polyposis in Intestinal cancer	21 years to 85 years, both sex	Ongoing	Randomized, double blind trial of Curcumin in treatment of intestinal adenomas in Familial Adenomatous Polyposis (FAP)	http://clinicaltrials.gov/show/NCT00927485
		Curcumin for oral mucositis in children receiving doxorubicin based chemotherapy	5–30 years, both sex	Ongoing (Phase III)	Randomized, single blind trial of curcumin for inhibiting Oral Mucositis in Children who are on Doxorubicin Based Chemotherapy. Dose: 5 ml	http://clinicaltrials.gov/show/NCT00475683
		To control advanced high-grade osteosarcoma	8–65 years, both sex	Ongoing (Phase I/II)	Non-randomized, open label trial for evaluation of Ashwagandha Root Powder Extract and Curcumin Formulation in the treatment of Advanced High-Grade Osteosarcoma	http://clinicaltrials.gov/show/NCT00689195
		To treat advanced pancreatic cancer	18 years and older, both sex	Ongoing (Phase II)	Open label trial to see if curcumin treatment can help slow or shrink the growth of pancreatic cancers. The action of curcumin on the functioning of pancreatic cancer cells and the treatment safety Dose: 8 g	http://clinicaltrials.gov/show/NCT00094445
		Curcumin in combination with gemcitabine in pancreatic cancer patients	18 years and older, both sex	Ongoing (Phase II)	Non-randomized, open label trial to see if curcumin can improve the efficacy of the standard chemotherapy gemcitabine in advanced pancreatic cancer patients. Dose: 8 g	http://clinicaltrials.gov/show/NCT00192842
		In treatment of metastatic colon cancer	18 years and older, both sex	Not yet open (Phase III)	Randomized, double blind trial that evaluates curcumin and celecoxib in combination with gemcitabine for colon cancer patients.	http://clinicaltrials.gov/show/NCT00295035
Andrographolide	*Andrographis paniculata* (Acanthaceae)	The safety and efficacy of Andrographolide in elderly patients with or without Capecitabine	65 years and older, both sex	Phase 2	Randomized, open label trial to evaluate the efficacy and safety of Andrographolides combined with Capecitabine in the treatment of elderly patients with locally advanced or recurrent or metastasis inoperable colorectal cancer. Dose : 500 mg	http://clinicaltrials.gov/show/NCT01993472
Ginsenoside Rg3	*Panax ginseng* (Araliaceae)	Safety and efficacy of ginsenoside Rg3 in Hepatocellular Carcinoma stage I and II	18–75 years, both sex	Completed	Randomized, double blind trial, to determine the safety and efficacy of ginsenoside Rg3 and placebo in prevention and treatment of postoperative recurrence of liver cancer, respectively. Dose : 20 mg	http://clinicaltrials.gov/show/NCT01717066
		In advanced gastric cancer	18–75 years, both sex	Phase2	Randomized, double blind trial to evaluate the Ginsenoside Rg3 safety in advanced gastric cancer, and its effect on improvement in the efficacy of first-line chemotherapy. Dose : 20 mg	http://clinicaltrials.gov/show/NCT01757366
Parthenolide	*Tanacetum parthenium* (Asteraceae)	Dose escalation study in cancer patients	18 years and older, both sex	Phase I	Non-randomized, open label trial was carried out to determine toxicity and pharmacokinetics of parthenolide. Dose : 1, 2, 3, 4mg	Curry et al., 2004
Thymoquinone	*Nigella sativa* (Ranunculaceae)	Toxicity in patients with hematological malignancies	18 years and older, both sex	Phase I	Non-randomized, open label trial was carried to evaluate anti-cancer effects of the drug in advanced cancer and to evaluate general toxicities. Dose : 3, 7, 10 mg	Al-Amri and Bamosa, 2009
Resveratrol	Red wine	Effect of resveratrol in patients with colon cancer	18 years and older, both sex	Completed	An interventional, randomized, open label clinical trial was carried out to determine the effects of resveratrol on the Wnt signaling pathway in a clinical trial of colon cancer patients. Dose: Plant-derived tablets: 80, 20 mg/day Grape powder: 120, 80 g/day	http://clinicaltrials.gov/show/NCT00256334
		In treatment of Adenocarcinoma of the Colon Adenocarcinoma of the Rectum stage I and stage II	18 years and older, both sex	Completed	Non-randomized, open label trial in patients with Colorectal cancer Patients to study its tolerability, Target Tissue Levels and Pharmacodynamics	http://clinicaltrials.gov/show/NCT00433576
		To treat Unspecified Adult Solid Tumor.	18 years and older, both sex	Ongoing (Phase I-II)	Interventional open label trial to study the side effects and best dose of resveratrol in inhibiting cancer in healthy volunteers.	http://clinicaltrials.gov/show/NCT00098969
Epigallocatechin-3-gallate	Tea, grapes and red wine	Evaluation of uateNCT the Chemopreventive Effects of Epigallocatechin gallate (EGCG) in Colorectal Cancer (CRC) Patients With Curative Resections	18 years and older, both sex	Ongoing	Randomized, controlled pilot trial of patients with histological documentation of rectal adenocarcinoma or primary colon with resectable cancer, who have not gone through any cancer treatments Dose : 900, 450 mg	http://clinicaltrials.gov/show/NCT02891538
Quercetin	Buckwheat, Ginkgo biloba.	In treatment of Adenocarcinoma of the Prostate Recurrent Prostate Cancer stage I, IIA,IIB,III and IV	40 to 75 years male	Ongoing (Phase I)	A Phase I Randomized, Double-Blind, Placebo-Controlled Two-Arm Study of Green Tea and Quercetin.	http://clinicaltrials.gov/show/NCT01912820
		In treatment of colon cancer	18 years and older, both sex	Completed	Randomized clinical trial to study the effectiveness of sulindac, curcumin, rutin, and quercetin in preventing colon cancer.	http://clinicaltrials.gov/show/NCT00003365
Apigenin	Onions, tea, parsley, wheat sprouts	In case of breast cancer	18 years and older, both sex	Ongoing	Interventional clinical trial studies to determine the best dose and side effects of apigenin in increasing health benefits in patients of high-risk breast cancer.	http://clinicaltrials.gov/show/NCT03139227
		In treatment of colon cancer	50–75 years, both sex	Ongoing (Phase II)	Randomized double blind clinical trial to evaluate that the dietary supplementation with bioflavonoids will inhibit the recurrence rate of colonic neoplasia. Dose : 20 mg	http://clinicaltrials.gov/show/NCT00609310
Indole-3-carbinol	Cruciferous vegetables	In case of breast cancer	18–70 years female	Completed	Interventional, open label trial to study the efficacy of I3C in preventing breast cancer in women who are nonsmokers and are at high risk for breast cancer.	http://clinicaltrials.gov/show/NCT00033345
		Unspecified Adult Solid Tumor	18–70 years, both sex	Completed	Randomized phase I trial is studying the best dose and side effects of I3C and to evaluate how well is it effective compared to placebo in inhibiting cancer in healthy participants.	http://clinicaltrials.gov/show/NCT00100958
Ellagic acid	Strawberries, blackberries, walnuts, and raspberries	In treatment of colorectal cancer	18 years and older, both sex	Completed	Randomized, single blind, trial of pomegranate extract formulations in colorectal cancer Patients to study metabolic and gene expression profiling in tumoral and normal Colon Tissues	http://clinicaltrials.gov/show/NCT01916239
Diindolylmethane	Cruciferous vegetables	Pharmacokinetics and safety of diindolylmethane.	18–70 years, both sex	Completed	Randomized, double blind clinical trials to study the side effects and best dose of diindolylmethane in preventing cancer in healthy volunteers.	http://clinicaltrials.gov/show/NCT00784394
		For breast cancer	35–52 years female	Completed	Randomized, double blind trial to study the efficacy of DIM Supplements to enhance 2-OHE1/16 Ratio in Premenopausal Mexican Women With Risk of Breast Cancer Dose : 75 mg	http://clinicaltrials.gov/show/NCT02525159

This book chapter provides complete account of the phytochemicals that show anticancer potential by inhibiting PI3K pathway. Many preclinical, clinical, *in-vitro* studies are being carried out worldwide to determine the efficacy of phytochemicals that inhibit PI3K pathway for cancer treatment. For some of the phytochemicals, in addition to studies on cell lines, clinical trials conducted have shown successful outcomes such as in the case of ellagic acid and indole-3-carbinol. For some of the other phytochemicals such as resveratrol, epigallocatein-3-gallate, apigenin, and quercetin, studies on cell lines have given promising results and the clinical trials for these phytochemicals are ongoing. But still, there are many phytochemicals which have the potential to successfully treat cancer by targeting PI3K such as luteolin, fistein, evodiamine, benzyl isothiocyanate. Research on such phytochemicals is need of the hour.

## Author contributions

VS: contributed toward conceptualization, planning and writing the paper. MM, TK, and PC: contributed toward data collection and writing the paper. PS: contributed toward editing of manuscript.

### Conflict of interest statement

The authors declare that the research was conducted in the absence of any commercial or financial relationships that could be construed as a potential conflict of interest.
